# About the journal

**DOI:** 10.4103/0972-124X.44083

**Published:** 2008

Dear Reader,

It is indeed a pleasure to present you with the Volume 12 Year 2008 issues of the Journal of the Indian Society of Periodontology.

The ISP is really fortunate to have a wealth of experienced academicians who have painstakingly reviewed and corrected the articles that the diligent, young and energetic minds across the country have toiled to bring to your purview. I do hope you will relish the scholarly fare.

I would like to particularly thank the Publishers, Medknow Publications and specifically Dr. D. K. Sahu, its Director for their painstaking work in the short time that we have had this year to bring out the 3 issues. Thanks to their efforts, the JISP is now online at www.jisponline.com. You can submit articles directly from www.journalonweb.com/jisp.

The President Dr Praveen Kudva and the Honorary Secretary Dr. M. M. Dayakar deserve a special mention here for their help and encouragement in bringing out the JISP this year. A special thanks to Dr. D. G. Pol for the persistent stimuli he provided is seeing all the three issues Jan, May and Nov 2008 released before this years annual conference at Chandigarh.

I welcome your comments and suggestions on the JISP and as its Editor, I am fully committed to bringing about a JISP that reflects the quality and character of professional excellence of periodontology in India and the world today.

Sincerely yours,

D. Arunachalam
Editor,
Journal of Indian Society of Periodontology
E-mail: smilesindia@gmail.com

